# Topical Application for Tumors of the Breast

**Published:** 1860-04

**Authors:** 


*Topical Application for Tumors of the Breast.*

There are some benign tumors of the breast, resembling cancer, which are frequently extirpated. Dr. Chabrely has published some abservations on certain forms of these tumors, that can be cured without an operation, although months of treatment are required, and frequent applications of the following powder:

| | | |
|---|---|---|
| R.—Amyli, | grammes 2.50 | |
| Pulv. Iodini, | gramme 0.50 | to gramme 1. |
| Morphiæ muriatis, | " 0.40 | |

This powder is spread on some wadding, and then kept in contact with the diseased part by means of a suspensory bag.—*Bulletin Gén. de Thérapeutique.* S.

